# The Relevance of Infant Outcome Measures: A Pilot-RCT Comparing Baby Triple P Positive Parenting Program With Care as Usual

**DOI:** 10.3389/fpsyg.2019.02425

**Published:** 2019-10-29

**Authors:** Lukka Popp, Sabrina Fuths, Silvia Schneider

**Affiliations:** Clinical Child and Adolescent Psychology, Faculty of Psychology, Ruhr-University Bochum, Bochum, Germany

**Keywords:** randomized-controlled trial, transition to parenthood, behavioral problem, excessive crying, parenting training

## Abstract

Problems in infant mental health such as the ability to experience, regulate, and express emotional states is expressed in behavioral problems like excessive crying, feeding, and sleeping problems. Parenting programs are popular but their effectiveness on infant mental health remains uncertain. Possibly, because recent studies have focused only on parental and parent–child measures while they miss to assess infant behavioral measures. The goal of this pilot study is to fill in this gap by assessing infantile behavioral measures. We investigated the acceptance and first indicators of efficacy of the universal group parenting training Baby Triple P (BTP) compared to a care as usual (CAU) control condition focusing on early behavioral problems. In all, 49 couples were randomly allocated to receive either eight BTP sessions before birth and per telephone after birth or to take part in CAU. Infant behavior was assessed with a diary and a structured diagnostic interview. Parental self-report measures of partnership quality and parental competence were assessed before birth, 10 weeks after birth and at 6-month follow-up. Since the parent training was conducted before the birth of the child, the child’s mental health could not be assessed before the parent training. Thus, for this variable no within measurement (pre–post) could be carried out and intention-to-treat analysis was not possible. However, a between group analysis comparing BTP against CAU took place to assess effectiveness of BTP on children’s mental health. Mothers and fathers rated the program as feasible and relevant. Results indicate significant group differences in crying behavior 6 months after birth to the benefit of the intervention group. No beneficial outcomes were found for feeding and sleeping problems in infants or partnership quality, parental sense of competence in parents. Due to an unrepresentative high level of education of the participating parents and the small sample size, these findings can be considered preliminary. Nevertheless, these results allow to further investigate the effectivity of BTP in large-scale clinical trials. Behavioral diaries or diagnostic interviews for early mental health problems should be routinely implemented in randomized controlled trials (RCTs) in order not to miss possible behavioral changes in infants.

**Clinical Trial Registration:** www.ClinicalTrials.gov, identifier NCT02313493.

## Introduction

The first year of life has long been recognized as fundamental to a child’s mental health development. Infant mental health is defined as “the young child’s capacity to experience, regulate, and express emotions, form close and secure relationships, and explore the environment and learn” (Zero to Three, 2001). Early problems such as feeding and sleeping problems as well as prolonged crying are related to negative short- and long-term consequences for the whole family system but especially for the infant. Short-term, feeding problems can lead to malnutrition in infants (St. James-Roberts and Halil, 1991), sleeping problems to reduced sleep quality and to poor mood and emotion regulation (Mindell et al., 2017). Prolonged crying is very distressing for infant and parents and is documented as the most common trigger for abusive head trauma (also known as shaken baby syndrome; Barr et al., 2006) and abusive fractures (Leventhal et al., 2010). Long-term, early behavioral problems are a significant predictor of mental disorders in later childhood. A meta-analysis showed that prolonged crying is associated with later problems in behavioral and emotional regulation indicated by higher rates of internalizing and externalizing problems in children who cried prolonged during infancy. Sleeping problems are associated with disturbed attention regulation in later childhood. Early sleeping problems are linked to externalizing problems, particularly ADHD in school age. In sum, prevention of early behavioral problems at the earliest point in time is vital (Hemmi et al., 2011).

To promote mental and physical wellbeing of parents and infants, preventive programs in early mental health care target different groups and objectives and are provided by different professional groups (nurses, psychologists, doctors etc.). They target on (1) intrapersonal wellbeing and competence (e.g., sense of parental competence) or on (2) the quality of interpersonal contact between parents and child (e.g., positive parenting strategies). The assessments vary with the underlying theory of the program. The focus of the present study lies on cognitive behavioral programs (for attachment-based programs see e.g., Riva Crugnola et al., 2016; Camoirano, 2017). Meta-analyses showed overall medium effects on parental wellbeing (*d* = 0.31) and parenting skills (*d* = 0.70) (Pinquart and Teubert, 2010; Mihelic et al., 2017). How these programs affect infant mental health outcomes is rarely assessed. From 36 studies in total across the last 35 years, sleeping behavior was assessed in only 13 studies and crying behavior in only 8 studies as Mihelic et al. (2017) reported in their meta-analysis. Feeding behavior is the least assessed behavior (e.g., Hiscock et al., 2014). So far, mixed results of early parenting programs on sleeping behavior have been found. Around one half of the studies had significant beneficial effects on sleeping behavior and the other half reported an overall small effect size (*d* = 0.24). For crying and feeding behavior, no conclusions can be drawn yet. The data on crying behavior could not be interpreted possibly due to low power, as the authors suggested (Mihelic et al., 2017). In sum, the effects of early parenting programs are unclear so far. These programs are primarily developed to prevent early mental health problems and to promote a good start in life. Nevertheless, it seems remarkable that infant measures are rarely assessed, especially in the first year of life. Certainly, infant behavioral measures are necessary to add important information on the efficacy of parenting trainings, in particular with respect to the benefit of these programs for the wellbeing of infants themselves.

A neglect of infant mental health has been demonstrated in recent randomized controlled trials (RCTs) of early parenting programs. Latest examples of cognitive behavioral based programs are the infant-adapted versions of the internationally widely accepted programs Triple P [Baby Triple P (BTP); Spry et al., 2010] or the Incredible Years Series; Incredible Years Parents and Babies Program (Pontoppidan et al., 2016). These programs are based on social learning, cognitive behavioral and developmental psychology theory and are adapted to the special needs of new parents. They target to promote positive parenting relationships between parents and their children by enhancing early parenting confidence and behavior, parental coping, and supporting the couple relationship. Even though these programs are focused on infant mental health, it is astounding that the trials did not assess changes in infant behavior (crying, sleeping, eating behavior). The main outcome variables were measures of (1) parental wellbeing, parenting confidence, (2) infant’s physical and emotional development, temperament, and (3) quality of mother infant interaction (e.g., Tsivos et al., 2014; Mihelic et al., 2017, 2018), but not infant mental health (e.g., regulation behavior problems).

The present study aimed to contribute to current research by providing empirical data on the efficacy of one of the promising early parenting trainings BTP on infant mental health outcomes as primary outcome variable. We hypothesized that infants in the BTP-group would show less problems in sleeping, feeding, and crying behavior compared to infants in the care as usual-group (CAU-group) at 6 months of age. Infant mental health problems (feeding, sleeping disorders, and prolonged crying) was categorically diagnosed by the use of a structured diagnostic interview and observed in detail through a behavioral diary. As secondary outcome variables, parental mental wellbeing and acceptance of the program were assessed. We expected that compared to parents receiving CAU, parents receiving BTP would report beneficial outcomes on the quality of partnership and parental self-efficacy after the training and at the 6-month follow-up. Small effect sizes were expected, as the present study focused on prevention rather than on treatment of clinically significant mental health problems.

## Materials and Methods

### Training Protocol and Trainer Preparation

Baby Triple P is a group training that aims to promote parenting skills to address commonly occurring developmental and behavioral difficulties in infants across the transition to parenthood up to 12 months after birth (Spry et al., 2010). The program is an element of a series of parenting trainings called Triple P – Positive Parenting Program^®^ developed by the Parenting and Family Support Centre at the University of Queensland, Australia. The program is provided in four face-to-face group sessions and four follow-up sessions conducted by telephone. BTP can be implemented as a universal prevention or as an early intervention program during pregnancy and/or after birth. In the present study, the program was provided as an early prevention program for healthy first-time parents in a community sample.

A workbook (Spry et al., 2010) provided detailed information about the development of infants, parenting skills, psycho-education for becoming parents about mental wellbeing and partnership as well as corresponding exercises. The group-sessions included (1) principles of positive parenting, (2) promoting infant development, (3) adjusting to challenges as a parent and (4) partner support. The phone-sessions included (5–7) implementing parenting routines and (8) maintenance of learned skills and closure of the program (see Table 1 for more information about session content). Two clinical psychologists who were trained and certified in Baby- and Standard Triple P delivered all sessions. They received supervision from a senior psychotherapist.

**TABLE 1:** Extract of session content of Baby Triple P.

**Session title**	**Content**	**Strategies**	**Aim**
Session 1: Positive parenting	• Information about positive parenting • Overview of factors that impact infants’ development • Goals for the first year of life	• Spending time with the baby • Communicate age appropriately • Showing affection	Developing a positive baby–parent relationship
Session 2: Responding to the Baby	• Strategies for responding to the baby and teaching new behaviors • Information about infants’ sleep and cry behavior • Managing infants’ distress and sleeping difficulties (implement sleeping routines)	• Settling techniques • Establish limits • Encourage contentment • Offer diversion • Flexible routines • Praise, spend attention	Responding age appropriately and encouraging desirable behavior
Session 3: Survival skills	• Common experiences of new parents (i.e., parenting traps) • Understanding and coping with negative emotions (automatic thoughts, ABC-model)	• Abdominal breathing • Social support • Personal coping plan with positive statements	Coping with high-risk situations
Session 4: Partner support	• Common experiences of couples when the baby arrives (i.e., partner traps) • Communication with the partner and partner support	• Communication skills • Task planning (household, baby care) • Talk about feelings and needs	Maintain relationship happiness
Session 5–8: Implementing parenting routines	• Individual telephone sessions to help parents implement parenting skills and routines (session 1–4) in daily life	• Slight prompting from the practitioner to promote self-regulation • Review the progress and set goals for the future	Maintain change

### Participants

A total of 49 becoming parents were randomized to the BTP- (*n* = 28) or CAU-group (*n* = 21, see Supplementary Appendix 1 for CONSORT diagram detailing flow of participants through the study). The sample of first-time parents facilitated to control for earlier parenting experiences with siblings. Thirty-six couples participated in the BTP- (*n* = 20) or the CAU-group (*n* = 16). One mother and one father participated by themselves without a partner. From 57% of the randomized participants, follow-up data were available (30 mothers, 25 fathers) for the final analyses. The relatively low rate of participation was caused by scheduling difficulties, pre-term births and medical problems due to pregnancy between randomization and the start of the training. The mean age was *M* = 31.79 (*SD* = 3.35) for mothers and *M* = 34.07 (*SD* = 4.32) for fathers. The majority of participants were German (98%), highly educated (90% A-level and above) and lived with their partner (94%). Table 2 shows complete participant demographics.

**TABLE 2:** Participant demographics in BTP and CAU condition at baseline.

			**BTP (*n* = 20)**	**CAU (*n* = 15)**
**Measures**			***M* (*SD*)**	***M* (*SD*)**
Mothers		Age	32.20 (3.74)	31.24 (2.79)
	Level of education	>10 school years	85%	94%
		<10 school years	15%	6%
	Ethnicity	German	100%	94%
		Other	–	6% (*n* = 1)
Fathers		Age	34.24 (4.50)	33.81 (4.20)
	Level of education	>10 school years	85%	93%
		<10 school years	10%	7%
	Ethnicity	German	100%	93%
		Other	–	7% (*n* = 1)
Relationship status (%)		Living together	100%	88%
		Separated	–	12%
Infants	Female		45%	44%
	Male		55%	56%

#### Participant Recruitment and Selection Procedures

Becoming parents were recruited via gynecologists (9%), hospitals (2%), midwives (23%), child health nurses (7%), pregnancy-related courses (e.g., yoga) (35%), advertisement in the newspaper or on the radio (23%). Participants were eligible for inclusion if they were becoming parents and had a basic level of German literacy to follow the research and treatment material. Participants were excluded if they reported medical pregnancy complications.

### Procedure

Before the independent randomization (throwing a dice), participants were screened for eligibility over the phone. Participants were informed about the study and gave their written informed consent. The signed informed consent forms were sent back by mail. Questionnaires were assessed by choice online with LimeSurvey©^1^ or as hard copy (6%). The Baby-DIPS diagnostic interview was conducted on the telephone. Interviewers were two female psychologists with Master degree in clinical psychology who also carried out the parenting program. Before the implementation, they completed a standard training about the use of the clinical diagnostic interviews of the DIPS family (Kinder-DIPS or DIPS; e.g., Margraf et al., 2017) and additionally about the use of the Baby-DIPS. The training consisted of the coding and re-coding of audiotaped interviews until consistent diagnostic agreement was reached on the diagnosis and severity level. Interviewers received regular group supervision as required to discuss questions, difficulties or diagnostic decisions. The diary was filled in hard copy and sent in by mail. The assessments took place before birth (baseline, T1), 10 weeks after birth (post-treatment, T2), and at 6-month follow-up (T3). No monetary incentive for taking part was proposed, however, small age-appropriate gifts, greeting cards, and a certificate were offered. The research was reviewed and approved by the Ethics Committee of the Faculty of Psychology at Ruhr-Universität Bochum on September 4, 2012 (Votum 036).

### Measures

#### Infants’ Mental Health Measures

##### Crying, sleeping, feeding

Infants’ behavior and related parental emotions and cognitions were administered at T3 with the Structured Diagnostic Interview for the Assessment of Behavioral Problems in Infancy (Baby-DIPS; Schneider and Wolke, 2007). Current and lifetime diagnoses of crying, sleeping, and feeding problems and related clinical severity ratings (0–8, ≥4 indicates clinical significance) were assessed based on parental report. Good to excellent inter-rater reliability on the levels of single and lifetime behavioral problems (*k* = 0.77–0.98) were found (Popp et al., 2016) and replicated in the present study (*k* = 0.89–1.00).

##### Pattern of infants’ behavior

Infants’ crying, sleeping and feeding behavior was recorded by parents using the Baby Diary at T3 (adapted from Barr et al., 1988). The overall amount and frequency of crying, sleeping and feeding behavior was logged over three sequent days. Crying was further divided into fussing, crying and inconsolable crying. Fussing was described as an irritable or unhappy state that is not quite crying (Barr et al., 1988). In contrast to crying being consolable and reasonable infant behavior (e.g., tired, hungry, bored), inconsolable crying was described as inconsolable crying bouts that occur unpredictably, starting and stopping for no obvious reason and clinically described as colic or excessive crying (Barr et al., 2014). Additionally, parents recorded their level of frustration with the crying of their infant for each day on a 6-point Likert scale ranging from 0 = not at all to 5 = extremely. Psychometric properties of the diary have only been published for infant sleep. Here, good to moderate agreement between parental recordings and the assessment of sleep duration with an actigraph was found (*r* = 0.47–0.70) (Müller et al., 2011).

#### Parental Measures

##### Participants’ acceptance of the parenting training

A 13-item questionnaire was adapted from the protocol for treatment integrity developed by Schneider et al. (2013) for the present study. Parents were asked on a 4-point Likert scale (1 = does not apply to 4 = applies exactly) whether the program was feasible and the content relevant to them. Eight items were positively and five items were negatively formulated. A good to excellent reliability was found both for mothers (α = 0.92) and for fathers (α = 0.88).

##### Psychopathological wellbeing

Psychopathological symptoms were administered with the Brief Symptom Inventory 18 (BSI-18; Derogatis, 2000) at T2 to control for mental health problems. The short version of the Symptom Checklist (SCL-90-R, Derogatis, 1977, 2000) contains three 6-item scales for somatization, depression, anxiety and a global score (GSI). In the present sample, good reliability was found for mothers (α = 0.84) and fathers (α = 0.91), comparable with the psychometrics found in a clinical sample (α = 0.79–0.84; Franke et al., 2011). Differently from stated on clinicaltrials.gov, the DASS-21 and EPDS scores are not reported in this manuscript. The DASS-21 seemed not valid for new parents. Depressive symptoms such as lack of energy or difficulties to relax are common experiences of new parents. The results of the EPDS are also left out due to a lack of comparability with paternal affect-related symptoms.

##### Partnership

The quality of partnership was assessed with the short form of the Partnership Questionnaire (PFB-K; Hahlweg, 1996) at all three time-points of measuring. This self-administered questionnaire has nine items on three subscales (conflict behavior, affectionateness, communication) on a 4-point Likert Scale plus an additional item assessing how happy participants are in their partnership (0 = very unhappy to 5 = very happy). Good values of internal consistency for the total scores of the PFB-K (α = 0.84) were reported, whereas acceptable values of alpha were found in the present study for mothers (α = 0.67) and fathers (α = 0.78).

##### Parenting

Parental self efficacy and satisfaction with parenting was assessed with the Parental Sense of Competence Scale (PSOC; Gibaud-Wallston and Wandersman, 1978, cited in Johnston and Mash, 1989). The 17-item questionnaire (6-point Likert scale) was not suitable during pregnancy at T1. Hence, no differences between the total scores before and after the training were assessed. Differences between the BTP- and the CAU-group were only measured after the training (T2) and at 6-month follow-up (T3). An acceptable level of internal consistency has been reported in earlier studies with infants and older children (range α = 0.75–0.88) (Johnston and Mash, 1989; Knoche et al., 2007; Troutman et al., 2012) and was replicated in the present study for mothers (α = 0.70) and fathers (α = 0.82).

### Treatment Integrity

Group and telephone sessions were video- or audiotaped. A checklist with the program specific elements (57 total items for group session, 22 items for telephone sessions) was prepared for the present study. 10% of the group training and 10% of the individual telephone sessions were randomly selected for the assessment of manual integrity. One trained research assistant coded whether each element was present in the selected session and whether elements outside of the manual were included. Additionally, we rated the level of support by the instructor for participants to take actively part during the group sessions and to talk about difficult topics during the telephone sessions on a 6-point scale (1 = very poor to 6 = very well).

### Statistical Analyses

Analyses were conducted with SPSS 22.0.0.0 for Mac. Missing data was deleted pairwise. To compare the outcomes of the BTP- and the CAU-group on the diary at T3, *t*-tests for independent samples were calculated. Differences between the number of behavioral problems (Baby-DIPS) in the BTP- and CAU-group were examined with chi-square tests with Cohen’s *w* coefficients as a measure of effect size indicating values of <0.1 small, >0.3 moderate, and >0.5 large effects (*w*; Cohen, 1988). In order to test the hypotheses that BTP was associated with favorable outcomes in parental mental health compared to CAU, mixed-model ANOVAs with Bonferroni correction were performed on the parent variables (BSI-18, PFB-K, PSOC) from baseline to post-treatment and to 6-month follow-up (if available). ANOVA is described as robust and stable even if the distribution of the dependent variable in each group is not normal in form (Maxwell and Delaney, 2004).

## Results

### Treatment Integrity

Within the program, 97% of the 57 (*SD* = 4%; range 80–100%) elements of the program were provided during the group sessions and 100% of the 22 (*SD* = 11%; range 54–100%) elements during the telephone sessions. The trainer’s engagement to support the participants to take actively part was rated as good to very good for the group sessions (*M* = 4.52; *SD* = 0.4%) and for the telephone sessions (*M* = 4.98; *SD* = 0.3%). In 14% (*SD* = 22%; range 0–75%) of the telephone sessions, at least one topic outside the manual was discussed (e.g., breast feeding).

### Infants’ Mental Health Measures

#### Crying, Sleeping, Feeding

Infant behavioral problems were assessed with the Baby-DIPS at the level of current and lifetime diagnoses. For the present sample, feeding problems, sleep onset problems, and excessive crying were evaluated. The criteria for sleep maintenance problems were not assessed because the criterion for age (older than 6 months) was not appropriate. Chi-square analyses indicated no significant between-group differences of the number of feeding problems (χ^2^ = 0.86, *df* = 1, *N* = 33, *p* = 0.35), sleep onset problems (χ^2^ = 0.44, *df* = 1, *N* = 33, *p* = 0.51), excessive crying and the total number of lifetime behavioral problems (χ^2^ = 0.55, *df* = 1, *N* = 33, *p* = 0.46).

#### Pattern of Infants’ Behavior

Based on the data of the diaries (see Table 3), *t*-tests for independent samples revealed a significant difference between infants whose parents participated in BTP and those whose parents did not participate, in the *frequency* of periods the infants were awake and content, *t*(25) = 2.29, *p* = 0.03, Cohen’s *d* = 0.88, and in the frequency of inconsolable crying, *t*(25) = 2.21, *p* = 0.04, Cohen’s *d* = 0.85. The mean number of frequencies indicated that infants of parents in the BTP-group were more frequently awake and content and cried less inconsolably during the three 24-h periods. No differences between the frequency of sleeping, feeding, crying, fussiness, and body contact, *t*(25) < 1.46, *p* > 0.16, Cohen’s *d* < 0.57 were found.

**TABLE 3:** Mean (*SD*) frequency and duration (min) of infant behavior and body contact between parent and infant in the BTP- and CAU-group 6 months after birth (T3).

	**Frequency/24 h**				**Duration (min/24 h)**			
	**BTP (*n* = 14)**	**CAU (*n* = 13)**	***t* (*df*)**	**Cohen’s *d***	**BTP (*n* = 14)**	**CAU (*n* = 13)**	***t* (*df*)**	**Cohen’s *d***
Sleeping	7.24 (2.01)	7.28 (2.24)	−0.53(25)	0.02	771.54 (44.80)	742.44 (62.32)	1.40 (25)	0.54
Awake and content	11.05 (4.06)	8.28 (1.60)	2.29(25)^*^	0.89	466.90 (72.47)	433.85 (68.28)	1.22 (25)	0.47
Fussy	7.52 (4.94)	5.52 (2.73)	1.32 (25)	0.50	80.54 (50.70)	81.67 (34.12)	−0.7(25)	0.03
Crying	1.15 (0.83)	1.92 (1.77)	−1.42(25)	0.56	11.55 (9.72)	25.00 (27.40)	−1,73(25)	0.65
Inconsolable crying	0	0.21 (0.35)	−2.21(25)^*^	0.85	0	3.59 (6.27)	−2.15(25)^*^	0.81
Feeding	6.97 (1.76)	6.79 (1.78)	0.26 (25)	0.10	102.97 (43.45)	128.21 (38.67)	−1.6(25)	0.61
Body contact	9.43 (4.86)	8.54 (3.82)	0.53 (25)	0.20	199.55 (140.13)	249.23 (113.49)	−1.02(25)	0.40

*Infant and parent behavior was assessed within a 24-h period on three successive days with a diary (adapted by Barr et al., 1988) when the infants were 6 months old. ^∗^*p* < 0.05.*

Between the CAU- and BTP-group, *t*-tests for independent samples revealed a significant difference between the *duration* (min) of inconsolable crying, *t*(25) = 2.15, *p* = 0.04, Cohen’s *d* = 0.81, and a marginal difference in crying, *t*(25) = 1.73, *p* = 0.09, Cohen’s *d* = 0.67. Infants in the BTP-group showed minimal less extended periods of inconsolable crying bouts (4 min) than infants in the CAU-group during the three 24-h periods. No significant group differences were found for the duration of sleeping, feeding, fussiness, crying, and body contact, *t*(25) < 1.59, *p* > 0.13, Cohen’s *d* < 0.79.

Parents in the BTP- and CAU-group reported similar patterns of sleeping at day and at night (10.00 am – 6.00 pm). Infants in the BTP-group slept 3.58 and in the CAU-group 3.77 times during the day, *t*(25) = 0.03, *p* = 0.74, *d* = 0.13, and awoke 1.81 (BTP) vs. 2.46 (CAU) times during the night, *t*(25) = 1.35, *p* = 0.19, *d* = 0.54. Parents in the BTP- (*M* = 0.52, *SD* = 0.65) and CAU-group (*M* = 0.91, *SD* = 0.70) both reported low mean levels of frustration about the crying behavior of their infants with no significant group-differences, *t*(25) = 1.49 *p* = 0.15, Cohen’s *d* = 0.58.

### Acceptance of BTP

#### Program Acceptance

Acceptance scores indicated that the parenting training was well accepted by mothers (*M* = 41.92, *SD* = 8.25, range 29–52) and fathers (*M* = 39.83, *SD* = 7.03, range 25–51). The possible sum score ranged from 13 to a maximum score of 52.

### Parental Measures

#### Psychopathological Wellbeing

The sum scores of the BSI-18 of the BTP- and the CAU-group after birth (T2) were below the cut-offs (for men ≥10; for women ≥13; Zabora et al., 2001) for clinically significant mental health symptoms. A *t*-test for independent samples showed no significant differences between the mean scores of the BSI-18 between the BTP- and CAU-group at T2 for fathers, *t*(26) = 1.68, *p* = 0.11, *d* = 0.68, and mothers, *t*(27) = 0.87, *p* = 0.43, *d* = 0.33.

#### Partnership

All parents showed PFB-K scores higher than the cut-off sum (<12; Kliem et al., 2012) for negative quality of partnership at all three time-points (BTP > 19, CAU > 18). For mothers, a 3 (time: T1, T2, T3) × 2 (group: BTP, CAU) mixed-model ANOVA revealed a significant main effect of time, *F*(2, 21) = 4.2, *p* = 0.03, η^2^ = 0.30, indicating that overall, PFB-K mean scores changed across the three time-points of measurement. Bonferroni corrected *post hoc* tests indicated that PFB-K scores did not change from T1 to T2 (*p* = 0.22) but from T1 to T3 (*p* = 0.01) and from T2 to T3 (*p* = 0.01), indicating that mothers reported higher quality of partnership at T1 (*M* = 2.39, *SD* = 0.44) compared to T3 (*M* = 2.21, *SD* = 0.51). There was no significant interaction between time and group, *F*(2,20) = 0.83, *p* = *0.45*, η^2^ = 0.08.

For fathers, a 3 (time: T1, T2, T3) × 2 (group: BTP, CAU) mixed-model ANOVA revealed no significant main effect of time, *F*(2,20) = 2.48, *p* = 0.11, η^2^ = 0.20, indicating that fathers showed no differences in the mean scores on the PFB-K across the three time-points. Analog to the mothers, there was no significant interaction between time and group, *F*(2,20) = 2.46, *p* = 0.11, η^2^ = 0.20.

#### Parenting

The mean sum scores of the PSOC across T2 and T3 were at the higher bound (*M* > 68) in both groups compared to a normative sample with parents of pre-school aged children (*M* > 63; Johnston and Mash, 1989), indicating an overall high sense of parental competence and satisfaction with the parental role in the present sample. A 2 (time: T2, T3) × 2 (group: BTP, CAU) mixed-model ANOVA revealed no significant main effect for time in the group of mothers, *F*(2,25) = 0.18, *p* = 0.67, η^2^ = 0.01 and fathers, *F*(2,19) = 2.59, *p* = 0.12, η^2^ = 0.12. This indicates that for mothers and fathers the parental sense of competence reached comparable values after the training and at 6 months after birth. Furthermore, there was no significant effect between time and group (BTP vs. CAU) for mothers, *F*(2,25) = 0.05, *p* = 0.83, η^2^ = 0.00 or fathers, *F*(2,19) = 0.00, *p* = 0.96, η^2^ = 0.00 in PSOC scores.

## Discussion

The present RCT-study investigated the efficacy of BTP compared with CAU in a community sample on early behavioral problems in infants. Additionally, acceptability of the program and parental outcomes (partnership quality, parental sense of competence) were investigated. The study extends previous research that has rarely assessed infant behavioral variables.

### Infants’ Mental Health Measures

#### Pattern of Infants’ Behavior

Crying was split up into three crying modes of fussing, crying (reasonable, consolable), and inconsolable crying bouts. A 3-day 24-h diary showed that parents in the CAU-group described the crying behavior of their infants as inconsolable (4 min/per day). In comparison, no inconsolable crying bouts were described in the BTP-group. And, infants in the BTP-group were described as more often awake and content. These differences were statistically significant but might be not clinically significant. In the sense that 4 min less intense crying a day must not necessarily result in a great relief for parents and children. At the same time, the frequency and duration of sleeping, feeding and body contact did not differ significantly. Similarly, the pattern of daily naps and awake periods at night did not differ significantly between the two groups.

One hypothesis that might explain these preliminary differences is the postulation that BTP provided parenting knowledge and skills that might have enhanced parental skills to regulate infant behavior, which in turn might have reduced inconsolable crying. Crying bouts are the most common problem in infancy with a heavy burden for parents and a high potential of an unfavorable developmental course for the child. Possibly, program elements that focused on coping with regulatory difficulties by recognizing infant needs, settling behavior and regulating one’s own emotional states were valuable. Additionally, parents in the BTP-group might have learned to interpret their infants’ crying as a temporal normal developmental behavior that they can cope with.

However, no beneficial outcomes for feeding and sleeping were found. Two explanations for these findings might be feasible. First, crying is more frequent during the first 6 months than difficulties in sleeping or feeding. In the first 3 months, crying is described as a behavioral state of normal development including prolonged and inconsolable crying bouts (Barr, 2004). The duration of crying increases from birth to 6 weeks of age (around 1.5–2.5 h per day) and declines at 4 months of age (1 h per day) (e.g., St. James-Roberts and Halil, 1991; Alvarez, 2004). For sleeping, a 24-h day and night rhythm is at the earliest established when the infant is 6 months old (Iglowstein et al., 2003). And, despite the fact that a high number of parents are concerned about feeding habits of their infant, clinically significant feeding problems are very scarce (3%; Eddy et al., 2015). Positive influence on crying was therefore more observable in the present study while sleeping problems become first evident after 6 months of age. Whether BTP can help to reduce sleeping and feeding problems should therefore be studied in a larger clinical sample with a follow-up measurement after 6 months of age when a day-night rhythm has most likely been established. The results of earlier studies support this idea. Around one half of the studies (5) that assessed sleeping behavior had no beneficial effects described in the meta-analysis of Mihelic et al. (2017). A closer look at the differences between trainings that were successful (*d* = 0.21–0.74) and those unsuccessful (*d* = 0.02–0.15) showed that the more effective trainings were targeted at infants with sleeping problems (e.g., initiating and maintaining sleep). The more ineffective trainings were targeted at infants in community samples without sleeping problems. This underlines the importance of target group specific trainings.

Another explanation might be that the diary used in the present study was more sensitive to changes in crying behavior than to feeding and sleeping habits. The diary provided a differentiated picture regarding the changes in frequency and duration of different modes of crying (fussing, crying, inconsolable crying) while problems in feeding (e.g., food refusal) or sleeping (e.g., sleep onset problems) were not operationalized in such detail. Further studies should also focus on the issue of feeding and sleeping patterns.

#### Behavioral Problems

Contrary to our expectations, infants in the BTP-group did not show less behavioral problems such as excessive crying, feeding- and sleep onset problems compared to infants in the CAU-group as assessed with a diagnostic interview (Baby-DIPS, Schneider and Wolke, 2007). Probably, the difference between infants in the two groups could not be indicated due to a low base rate of clinical problems in the present sample. Here, further research with a larger sample investigating the impact of BTP on behavioral problems would be of clinical value.

### Programs’ Acceptance

The program was highly accepted by mothers and fathers, indicated by favorable ratings on the acceptance questionnaire, concordant with previous findings (Ferrari et al., 2011; Tsivos et al., 2014). This suggests that the content and delivery mode (group- and couple-based before and after birth) of BTP was acceptable to the present sample and sensitive to the parents’ needs. The high acceptance of the program assists in the further development of BTP and in the implementation of future RCTs.

### Partnership

We hypothesized that parents in the BTP-group would show more favorable outcomes on psychosocial measures. This expectation was not confirmed. All parents reported a high quality of partnership across all three time-points. Overall, parental scores of partnership quality were lower 6 months after birth than before birth. However, this effect of time was only significant for mothers. These findings are in line with earlier longitudinal outcomes showing that partnership satisfaction deteriorates when an infant arrives (Belsky and Rovine, 1990) and that this phenomenon varies by gender (Nomaguchi and Milkie, 2003). Here, becoming mothers were found to be less satisfied due to more housework and conflicts with their partners. Based on these studies, the present results indicated that BTP did not alter the typical decrease of partnership quality during transition to parenthood.

### Parenting

Contrary to our expectations, the overall sense of parental competence and satisfaction did not differ between groups. A recent study showed that parental self-efficacy in mothers increased instinctively after birth in a non-clinical sample (Kunseler et al., 2014). In the present sample, the scores of parental self-efficacy were at the upper bound of a normative sample (Johnston and Mash, 1989). It can therefore be assumed that BTP could not additionally promote parenting competence due to a ceiling effect. In other words, parental sense of self-efficacy was already very high in all parents and could not be improved further by the BTP.

Limitations pertaining statistical power and methodological difficulties must be mentioned. First, the sample size was small (*N* = 49). The results must be therefore considered preliminary. Second, the present sample (e.g., highly educated, middle-aged, very low level of risk factors) is not representative with regard to socio-demographic status. Third, parental mental health wellbeing was not assessed at the first time-point of measurement due to technical problems. Depressive mood were not reported due to ambiguous descriptions of symptoms that commonly occur during the early phase of parenthood. Subsequently, the experimental hypotheses that parents in the BTP-group show less mental health problems than in the CAU-group could not be thoroughly investigated. Fourth, according to the current state-of-the-art, an attention placebo group as control condition should have been used in the present study to test the specific effect of the parenting training (Vickers and de Craen, 2000). However, the attempt to implement a moderated discussion group as attention placebo control arm was abandoned due to methodological and ethical problems (for a further discussion see Popp and Schneider, 2015).

## Conclusion

This pilot RCT represented one of the first applications of a group BTP that was delivered before and after birth in a non-clinical sample of becoming first-time parents focusing on infant-related mental health outcomes. Mothers and fathers accepted the program very well. Their expectations and need for information and support were met. As in other RCT’s of behavioral parenting trainings (Chacko et al., 2016), the rate of enrollment was low. As in all BTP studies run so far, no beneficial effects were found for parental outcomes. The use of a behavioral diary was suitable to determine differences in infant crying behavior. The assessment of infant mental health measures such as a diagnostic interview and behavioral measures, for example behavioral diaries, should routinely be implemented. Additionally, the assessment should be more tailored to measure distinctive parental skills matching infant needs at their various specific developmental stages. Currently, parental competence and parenting skills are assessed globally. However, each developmental phase requires a different repertoire of parental skills.

We concluded that the assessment of observable infant behavior provides relevant information about the efficacy of early interventions. This pilot study with all its limitations afford to examine the effect of BTP on early behavioral problems in large-scale RCT in a clinical sample. A special focus should lie on excessive crying as the major concern and source of stress for parents during transition to parenthood, which is also associated with infant maltreatment (Barr et al., 2006, 2014).

## Data Availability Statement

The datasets generated for this study are available on request to the corresponding author.

## Ethics Statement

The studies involving human participants were reviewed and approved by the Ethics Committee of the Faculty of Psychology, Ruhr-Universität Bochum. The patients/participants provided their written informed consent to participate in this study.

## Author Contributions

SF and LP provided the parenting training and conducted the research. LP analyzed the data and drafted the manuscript while SS and SF provided the critical feedback. All authors designed the study, read, and approved the final version of the manuscript.

## Conflict of Interest

The authors declare that the research was conducted in the absence of any commercial or financial relationships that could be construed as a potential conflict of interest.

## Footnotes

1 https://www.limesurvey.org
