# Supplementary material for: The Relevance of Infant Outcome Measures: A Pilot-RCT Comparing Baby Triple P Positive Parenting Program With Care as Usual
Source: Front Psychol. 2019 Oct 29;10:2425. doi: 10.3389/fpsyg.2019.02425 (PMC6828945; doi:10.3389/fpsyg.2019.02425)
Supplement: Supplementary file 1 [file Table_1.docx]

Appendix 1

Assessed for eligibility (60 couples, incl. 1 mother and 1 father without their partners)

Randomized (49 couples)

**Lost to post assessment**  (2 mothers, 3 fathers)
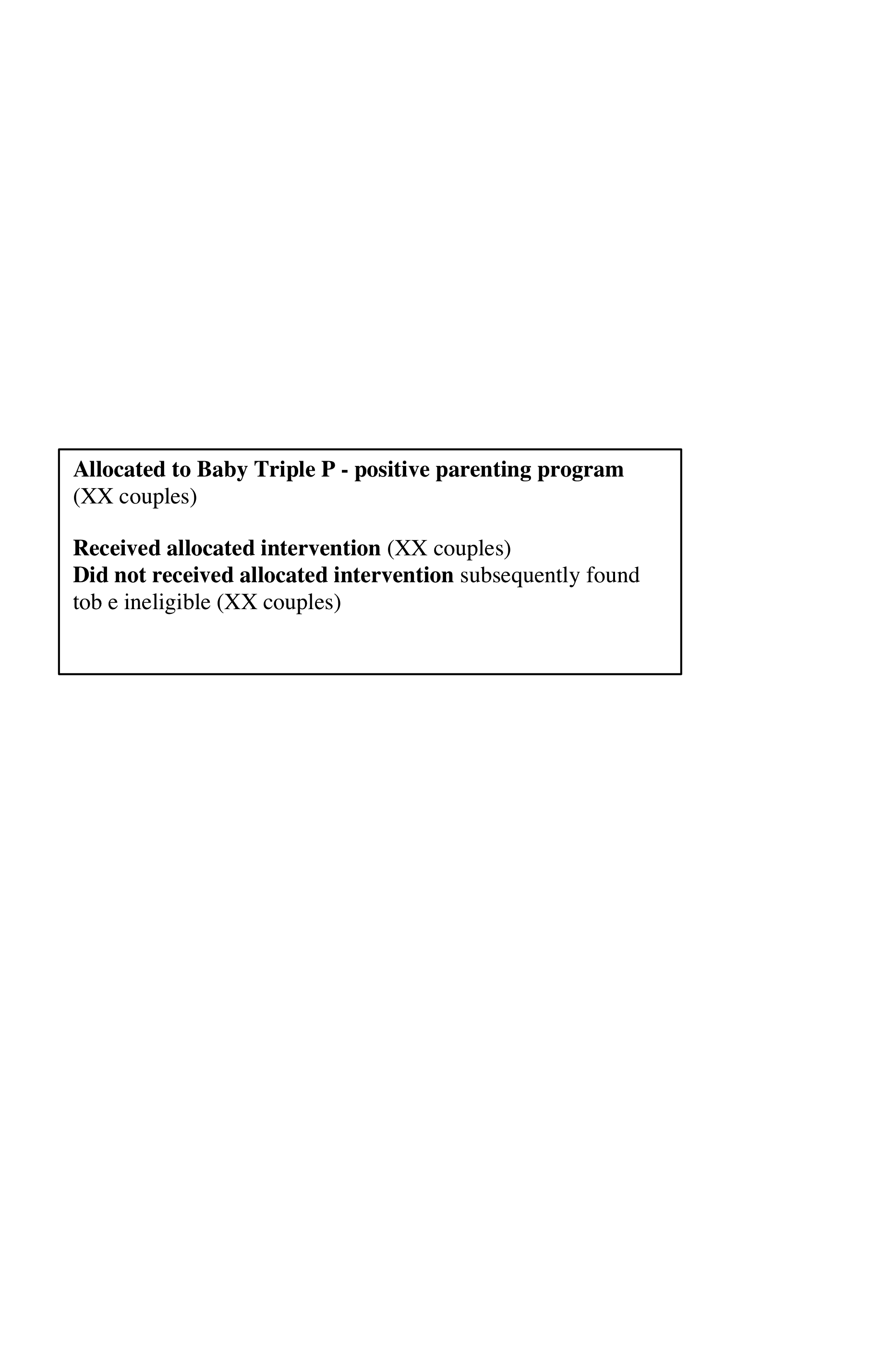


% did not respond toXXX

**Lost to post assessment**  (3 mothers, 5 fathers)

% did not respond toXXX

**Allocated to care as usual control group** (21 couples)

**Participated in control group** (16 couples, incl. 1 mother and 1 father without their partners)

**Did not participate in control group** (5 couples)

**Allocated to Baby Triple P - positive parenting program** (28 couples)

**Received allocated intervention** (20 couples)

**Did not received allocated intervention** dropped out due to scheduling difficulties (7 couples) and preterm birth (1 couple)

**Lost to follow up** (1 mother, 2 fathers)

**Lost to follow up** (no datasets were lost)

% did not respond toXXX

**Datasets analyzed** (16 mothers, 13 fathers)

% did not respond toXXX

**Datasets analyzed** (14 mothers, 12 fathers)

% did not respond toXXX

Excluded (did not met inclusion criteria/refused to participate 11 couples)

Participant flow chart detailing participant involvement through study process following Consolidated Standards of Reporting Trials guidelines.
